# Effects of Lyophilization on the Release Profiles of 3D Printed Delivery Systems Fabricated with Carboxymethyl Cellulose Hydrogel

**DOI:** 10.3390/polym13050749

**Published:** 2021-02-28

**Authors:** Xuepeng Jiang, Yanhua Huang, Yiliang Cheng, Zhan Zhang, Xiaolei Shi, Hantang Qin

**Affiliations:** 1 Department of Industrial and Manufacturing Systems Engineering, Iowa State University, Ames, IA 50011, USA; xuepengj@iastate.edu (X.J.); yanhuah@iastate.edu (Y.H.); 2 Department of Food Science and Human Nutrition, Iowa State University, Ames, IA 50011, USA; ycheng8@iastate.edu; 3 Center for Nondestructive Evaluation, Iowa State University, Ames, IA 50014, USA; zhan@iastate.edu

**Keywords:** 3D printing, carboxymethyl cellulose, hydrogel, lyophilization, dissolution, release model, customization

## Abstract

Recently, increasing numbers of researchers are becoming interested in 3D bioprinting because it provides customizability and structural complexity, which is difficult for traditional subtractive manufacturing to achieve. One of the most critical factors in bioprinting is the material. Depending on the bio-applications, materials should be bio-inert or bio-active, non-toxic, and along with those characteristics, mechanical properties should also meet the applicational or manufacturing requirement. As previously validated for bioprinting, carboxymethyl cellulose (CMC) hydrogel is focused on the printability and release control test in this study. With a differentiated weight percentage of CMC hydrogels were used to 3D print capsules filled with food degradable colorant at designated voids to mimic capsules manufactured for oral delivery. Standard USP (United States Pharmacopeia) dissolution apparatus II (Paddle) evaluations were performed both on lyophilized and non-lyophilized printed capsules. The first-order model was selected due to high linear fitting regression. Upon 24 h dissolution, non-lyophilized capsules showed a different release efficiency when the CMC percentage varied, while lyophilized capsules showed no significant difference. This study signifies the possibility of customizing oral drug delivery by printing capsules with CMC hydrogel. The improved delivery efficiency demonstrated by capsules with post-process lyophilizing proposed potential optimization options for pharmaceutical manufacturing industries.

## 1. Introduction

3D printing is an additive manufacturing process that fabricates three-dimensional CAD (computer-aided design) objects in a layer-by-layer buildup subsequence. Although the 3D printing idea came out in the 1980s [1], researchers in the engineering area still pay close attention to the 3D printing technique. Its advantage of high precision, controlling deposition, cost-effectiveness, simple processing, and fast prototyping compare favorably with traditional subtractive manufacturing in recent years [2]. Nowadays, the 3D printing technique involves interdisciplinary areas that include not only manufacturing but also civil construction, fashion and design, tissue engineering, pharmaceutical, and food production [2,3,4,5,6]. Material jetting (including drop on demand (DOD), nanoparticle jetting (NPJ) and material jetting (MJ)), powder bed fusion (including selective laser sintering (SLS), and selective laser melting (SLM)), directed energy deposition (DED) and material extrusion (ME) or semi-solid extrusion (SSE) are four major branches made up of 3D printing technology [7]. Among all additive manufacturing technologies, ME or SSE, FDM (fused deposition modeling), inkjet, and polyjet printing methods are commonly used in the bioprinting field [8,9].

3D bioprinting is of tremendous interest to researchers due to the need for high customizability for organ and tissue engineering [10] and wound therapy [11,12]. The biomaterial is the most crucial carrier to achieve an additive manufacturing 3D structure for bioprinting. Materials that meet the requirement of not inducing inflammation and allergy symptoms, non-toxic, biocompatibility or biodegradation, biofunctional, bioactive, bioinert, and sterilizable could be called biomaterials [13,14]. The most common biomaterials used in pharmaceutical and medical areas include metals, polymers, ceramics, and composite [15]. However, biopolymers have the largest applications because of their properties. In addition to the properties that biomaterials have, biopolymers also have some distinctive properties: flexibility (composition and shape of the form), resistance to biochemical attack, and light weight [15]. Among all biopolymers, carboxymethyl cellulose (CMC) is the most abundant native polymer on the earth, a renewable resource. CMC can easily form an aqueous hydrogel below 37 °C [16]; its properties brought tremendous interest to researchers for 3D bioprinting. Habib et al. studied the printability of alginate-carboxymethyl cellulose hybrid hydrogel and 3D printed scaffolds to investigate the viability of pancreatic cancer cell culturing [17]. Janarthanan et al. investigated the printability of hyaluronic acid–carboxymethyl cellulose hybrid hydrogel for soft tissue and organ regeneration with three different compositions by 50-layer height in complex structures without support materials or any post-processing. In their study, texture profile analysis, morphological analysis, in vitro cytotoxicity, and mice studies were conducted to indicate that the self-crosslinking capacity, composition, and other factors will have huge impacts on the generation of multilayered constructions for soft tissue engineering [16]. Kageyama et al. validated the feasibility of fabricating perfusable vasculatures using in situ cross-linkable gelatin–CMC hybrid hydrogels with cell embedding [18]. Calcagnile et al. composited polydimethylsiloxane (PDMS) and CMC to improve its hybrid hydrogel tactile properties and simulated the slimy effect of organic in the human body [19]. Pasqui et al. researched bone tissue scaffolds using hydroxyapatite–CMC hybrid hydrogel [10]. Ahlfeld et al. investigated the printing fidelity of alginate–CMC hybrid hydrogel as well with two different concentrations. Moreover, cell viability was tested with immortalized human Mesenchymal Stromal Cells (hMSCs) over 21 days of in vitro culturing [20]. Maver et al. used an polyethylene oxide (PEO) –CMC hybrid hydrogel with a spin-assisted layer-by-layer coating process to develop the wound dressing materials [11]. With different composites, carboxymethyl cellulose hybrid hydrogels could present specific features to meet bioprinting’s requirements.

Most researchers studied a 3D printing CMC hydrogel composite (such as composite with alginate, hyaluronic acid, gelatin, PDMS, and hydroxyapatite) in tissue engineering and wound dressing area [10,11,12]. Only a few researchers did investigate the release profile for their wound dressing materials, such as Ahlfeld et al. who studied the incorporation of synthetic nano-silicate clays with alginate–CMC hybrid hydrogel, which further increased the ability of the alginate–CMC samples to release loaded drugs in a more sustained manner [20]. Maver et al. tested PEO–CMC hybrid hydrogel 3D printed scaffold with a pain reliever, prolonging its efficacy for two days upon changing the dressing [11]. Few studies have investigated the food colorant acting as a dye for the drug delivery system during the dissolution test. Erythrosine (red-3) and Allura Red AC (red-40) were commonly used around the world. Red-3 can be used in colored food and ingested drugs; however, red-3 cannot be used in cosmetics and external drugs [21]. Thus, a red food degradable colorant (consists of red-3 and red-40) was chosen in this study for the in-fill material not only because of easily eye-observing during the printing and dissolution test but also because red-3 and red-40 can be used in the food and ingested drugs according to FDA approval as an edible dye.

In the pharmaceutical field, lyophilization has typically been used for anti-infectives, biotechnology-derived products and in-vitro diagnostics. Lyophilization or freeze-drying is a low-temperature dehydration process without passing through a liquid phase that involves freezing the product, lowering the pressure, then removing the ice by sublimation. However, the lyophilization post-processing costs time and resources to achieve long-term room temperature storage compared with non-lyophilization. Hence, this study focused on the effect of lyophilization on the dissolution test. A pre-study was conducted which found that carboxymethyl cellulose aqueous (mixed with DI water) hydrogel could be the potential printing material by adjusting the CMC concentration. This study used degradable food colorant and lyophilization post-processing to verify the release profile with three different CMC aqueous hydrogel concentrations.

## 2. Materials and Methods

### 2.1. Materials and Preparation

Carboxymethyl cellulose hydrogel was formulated with CMC 6000 Fine Powder (curtesy of Ticalose, White Marsh, MD, USA). The CMC to total aqueous hydrogel ratio was narrowed down from 8% to 12% (*w*/*w*) based on our preliminary experiment with a broader concentration range of 4–16% (*w*/*w*). CMC hydrogel below 8% showed insufficient mechanical integrity withholding the printing dimension, while above 12% demonstrated difficulties during the extrusion when it printed with a tip nozzle of 0.672 mm in diameter. The aqueous CMC hydrogels with concentrations of 8%, 10%, and 12% (*w*/*w*) were prepared as follows: 4, 5, and 6 g of CMC powder were mixed and homogenized with 44, 45, and 46 g of deionized water to formulate of 8%, 10%, and 12% (*w*/*w* in total solids) aqueous hydrogels at room temperature (24 °C), respectively; then, all samples were centrifuged at 3000 rpm for 10 min for air bubble removal. Food degradable colorant was purchased from Wilton (Naperville, IL, USA). The red colorants combined with red-3 (Erythrosine, C_20_H_6_I_4_Na_2_O_5_) and red-40 (Allura Red AC, C_18_H_14_N_2_Na_2_I_8_S_2_) was chosen as the in-fill material. Due to the pressure of inert thickener glycerol, in the red colorant formulation, the stock colorant was diluted ten times with DI water. All other chemicals were purchased from Fisher Scientific (Hampton, NH, USA) and used as they were received, unless specified otherwise.

### 2.2. Rheological Properties

The rheological tests were carried out using a Discovery HR-2 Rheometer (TA Instruments, New Castle, DE, USA) with parallel plate geometry at a gap size of 1 mm at 23 °C. The flow ramp test was conducted to determine the apparent viscosity under the increased shear rate (0.1 to 50 1/s). The oscillatory frequency sweep test was performed to characterize the materials’ dynamic modulus with 0.1–600 rad/s angular frequency increases. The rheological properties, i.e., shear rate, apparent viscosity, angular frequency, storage modulus (G′), loss modulus (G′′), and loss tangent (tan δ = G′′/G′) were recorded. All rheological properties tests were conducted in triplicate.

### 2.3. 3D Printing

**Figure 1:**
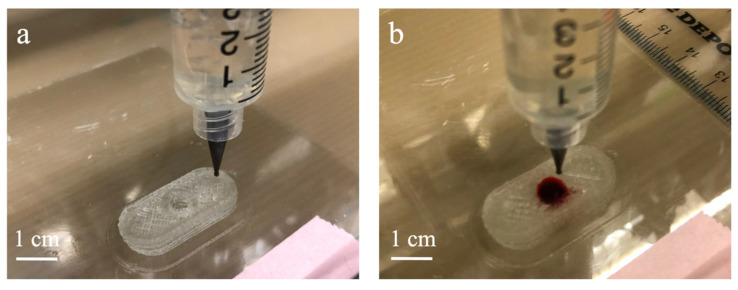
Printing process: (**a**) 7th layer; (**b**) colorant injected after the 7th layer.

Based on an FDM printer, K8200 (Velleman, Inc., Gavere, Belgium), a syringe holder add-on kit K8205 (Velleman, Inc., Gavere, Belgium) was used to allow the FDM printer to print semi-solids. For quality control, the capsule model was designed with ten layers in total, meaning the layer height was equal to the nozzle size. The first and last three layers were the enclosure. A circular void section with a diameter of 6.72 mm was printed from layer 4 to layer 7 to form a cylindrical hollow section for filling the colorant. Diluted red food degradable colorant was added to the capsule’s void section during the printing process. To be more specific, 20 μL of 10-fold diluted colorant was injected into the void section after the printer finished the 7th layer of the CMC aqueous hydrogel (See Figure 1a) and before the 8th layer enclosed the void section (See Figure 1b). The Cura-Engine was used for the 3D model slicing and G-code generation. 3D printing parameters were set as follows: the printing speed was 2.4 mm/s for the outer wall and 4 mm/s for the in-fill of 4% with the extrusion multiplier of 0.125.

### 2.4. Lyophilization

Lyophilization (also known as freeze-drying) post-processing was performed by freeze-dryer (Virtis Genesis SQ freeze-dryer, Gardiner, NY, USA) for 12 h at −45 °C in the condensation chamber under a low vacuum (below 2.6 × 10^4^ Pa). After 3D printing, the lyophilized samples were placed in the freezer at −20 °C with at least 4 h to wait for the samples to be fully frosted before lyophilization. The non-lyophilized samples were kept in the fridge at −20 °C until the lyophilized samples were taken out from the freeze-dryer. All samples were kept at room temperature (24 °C) 30 min before the dissolution test for a stable temperature.

### 2.5. Dissolution Test

The dissolution test was conducted following the USP apparatus II (Paddle) procedure (37 °C, 100 rpm) using a DT 126 light dissolution tester (Erweka, Hessen, Germany) in a time as mannerly as the 1, 2, 3, 4, 6, 8, 12, 24 h time points. For the first two hours, the samples were dissolved in the pH = 1 environment with 500 mL of 0.1 M HCl and switched to a pH = 7 environment with 500 mL phosphate buffer (K_2_HPO_4_ and KH_2_PO_4_). At each time point, four 100 μL samples were acquired and stored in a 96-well plate with a lid in the dark 4 °C fridge waiting for plate reading. The amount withdrawn was not replaced by a fresh medium. This experiment was performed in triplicate, and the mean value was taken. Blank and standard curves of the colorant were also stored on the same plate for each set.

### 2.6. Data Analysis

In this study, the zero-order equation, first-order equation, and Higuchi equation were selected to verify the release kinetic as these release models were used to determine the release kinetic, which is prominent nowadays [22].

Zero-order equation:

(1) $$Q_{t}=Q_{0}+K\cdot t,$$

based on the linear fit [22,23,24], here was simplified with:

(2) Y=A·X+B,

where *Y* is the cumulative release level, *X* is time in hour. First-order equation:

(3) $$\frac{dC}{dt}=-K\cdot c,$$

(4) $$\log\left(C\right)=\log\left(C_{0}\right)-\frac{K\cdot t}{2.303}$$

based on logarithm fit [22,23,24]. Here was simplified it with:

(5) $$Y=A-e^{\left(-B\cdot X\right)},$$

Higuchi equation is the most prominent model used in nowadays release profile. It was defined as

(6) $$f_{t}=Q=A\cdot \sqrt{D\cdot \left(2\cdot C-C_{s}\right)\cdot C_{s}\cdot t},$$

and which can be simplified in most cases as [22,23,24],

(7) $$Y=A\cdot X^{0.5}.$$

Nelder–Mead Simplex algorithm was chosen for use in SciDAVis for fitting the release model, and Origin 2019 (Northampton, MA, USA) was used for analyzing the cumulative release level. Standard curves for red-3 and red-40 were statically evaluated by linear fit with a concentration weight factor. All experiments were performed in triplicate.

## 3. Results

### 3.1. Rheological Properties

**Figure 2:**
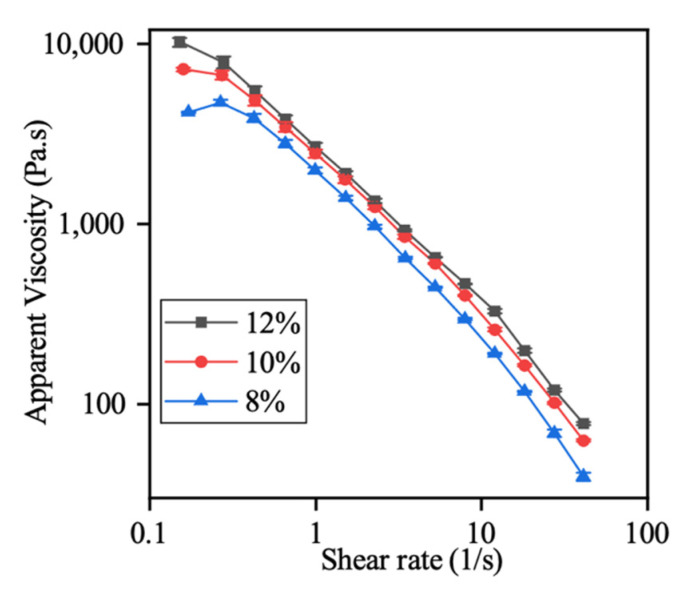
Apparent viscosity versus shear rate profiles of carboxymethyl cellulose (CMC) at different concentrations (8%, 10%, 12% *w*/*w*). Values are presented as the mean ± SD (*n* = 3).

As shown in Figure 2, CMC hydrogels’ apparent viscosity curves were plotted at three different concentrations. Regardless of the CMC concentration, the hydrogels demonstrated shear-thinning behavior, i.e., as the shear rate increased, the apparent viscosity decreased. The shear-thinning behavior of the CMC hydrogel is beneficial for semi-solid extrusion-based 3D printing. The shear rate-dependent viscosity could make material flowable during extrusion and hold the printed shape once the stress is removed [25]. In addition, the CMC hydrogels’ apparent viscosity increased as the concentration increased, which could be explained by the increased content of CMC provide more hydroxyl groups and results in more hydrogen bonding [26]. Compared with low concentration, a high CMC hydrogel concentration was associated with stronger mechanical integrity and shape retention ability due to the relatively high apparent viscosity. However, the low viscosity would make the low-concentration hydrogel easier to be extrude through the nozzle [17,26].

**Figure 3:**
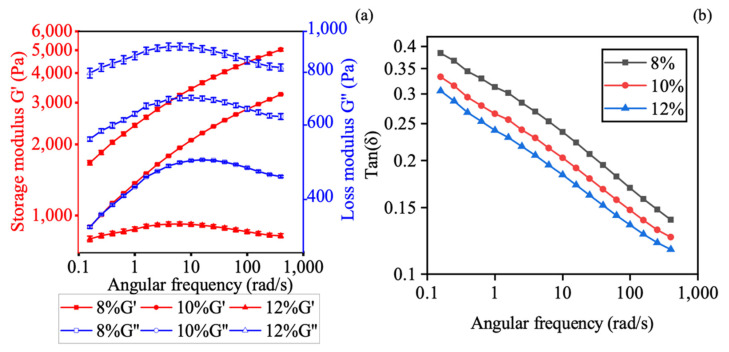
Oscillatory frequency sweep profile of CMC at different concentrations (8%, 10%, 12% *w/w*): (**a**) storage modulus (G′) and loss modulus (G′′); and (**b**) tan δ. Values are presented as the mean ± SD (*n* = 3).

Figure 3 shows the storage modulus (G′), elastic modulus (G′′) and phase angle (tan δ) as a function of angular frequency for the oscillatory frequency sweep test. The elastic modulus G′ and G′′ represent the ability of energy storage or dissipation via the polymer, respectively [27]. Tan δ is the ratio between G′′ and G′, which are less than one no matter the concentration of CMC hydrogel. This indicated that the G′ is always higher than G′′ during the frequency sweep test. Since the value of tan δ is less than one and G′ is higher than G′′, the CMC hydrogel showed viscoelastic solid-like characteristics rather than liquid-like properties [27]. The highest concentration of CMC hydrogel, 12% *w*/*w*, showed the highest G′ and lowest tan δ at the same angular frequency when compared with the lower concentration. The higher G′ indicated that the material has better shape retention ability after extrusion, and the lower tan δ revealed more solid-like behavior and poor fluidity [28,29].

### 3.2. 3D Printing and Lyophilization of CMC Aqueous Hydrogel

In this study, CMC aqueous hydrogel’s printability was defined into two categories: (1) hydrogel ink should be continuously and uniformly extruded out through a nozzle tip with the desired diameter; (2) the hydrogel ink should possess sufficient mechanical strength to support its own weight and minimize deformation after printing. However, high mechanical strength usually requires high viscosity and yield stress; high viscosity materials usually block the nozzle tip when extruding, thus resulting in a printing failure [2]. Our preliminary study found that the high-concentration (14%, 16%) CMC hydrogels resulted in blocking the 0.672 mm nozzle tip. The low concentration (4%, 6%) CMC hydrogels resulted in deformation during printing because of a lack of mechanical strength given the printing dimensions. Furthermore, liquid-like samples (printed with 4% and 6% CMC hydrogel) could not be easily peeled off from the glass substrate due to low viscosity, which increased the manufacturing failure rate.

**Figure 4:**
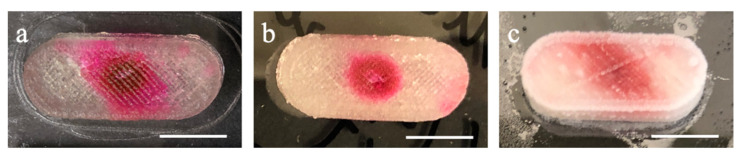
Capsule printed using: (**a**) 8%; (**b**) 10%; and (**c**) 12% CMC hydrogels (non-lyophilized, scale bar in 1 cm).

**Figure 5:**
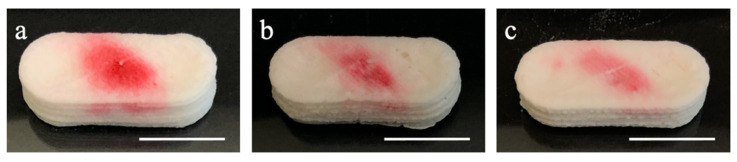
Capsule printed using: (**a**) CMC 8%; (**b**) CMC 10%; and (**c**) CMC 12% (lyophilized, scale bar in 1 cm).

Based on the preliminary study result, 8%, 10%, and 12% CMC hydrogels with printed samples (lyophilized and non-lyophilized) were chosen to be evaluated by the dissolution tests. Moreover, 8%, 10%, and 12% samples (Figure 4a–c, respectively) showed excellent retention after SSE printing. Moreover, they could be easily peeled off from the glass substrate without using any tools. Among the three concentrations, 8% CMC aqueous hydrogel presented the highest transparency. The higher the concentration is, the hydrogel became opaquer due to the higher polymer content. CMC shows amphiphilic characteristics as containing a hydrophobic polysaccharide backbone and many hydrophilic carboxyl groups [30]. Hence, as red-3 and red-40 are hydrophilic, the red colorants started diffusing when added to the capsule’s void section. The red colorants showed different diffusion degrees from Figure 4 and Figure 5 due to the colorant being hydrophilic and the different degrees of water content in each capsule; it started diffusing inside the capsule when the colorant was deposited. When the concentration increased, the diffusion level became less (as can be seen from both non-lyophilized samples and lyophilized samples).

Prior to the lyophilization post-process, the samples were frozen in a −20 °C freezer for 24 h. Subsequently, the samples were lyophilized at −40 °C for 24 h for dehydration. Compared to the non-lyophilized samples, the lyophilized samples consist of different amounts of CMC and the same amount of colorant (red-3 and red-40) in the capsule without moisture. Compared with samples without lyophilization, lyophilized samples require additional water to swell when placed into the solution. As shown in Figure 5, SSE-printed CMC hydrogel layers did not split or separate by hand, which means the attachment between layers by layers is stickiness or adhesiveness to make the capsules still retain their shape after lyophilization post-processing. Moreover, the lyophilization post-processing retains the capsule’s shape without significant dimension reduction (less than 5%). The red colorant showed a parallelogram pattern on the top surface because the colorant solution showed the capillarity following the tool path. The trivial gap between the filaments formed a tube-like structure provides the capillarity. The adhesion from the tube (trivial gap here) to the water (colorant solution here) let the solution keep going until the surface tension cannot hold its weight. In addition, the printing speed and feed rate did not match with each other—the feed rate is faster than the printing speed as a solid filament was wanted so the gap between the filaments can be kept under minimum air bubble—so the extruded filament was whirled in the void section.

## 4. Discussion

Release kinetics is an essential parameter of a drug delivery system as it indicates its efficacy and stability. An early burst release is sometimes preferable as giving the maximum relief instantly, followed by a sustained release to prevent repeated administration. Such as practical anti-cancer treatment demands the sustained release of the drugs [31]. Release mechanisms include a dissolution-controlled system (encapsulation), diffusion-controlled system (reservoir), dissolution and diffusion mixed controlled system (matrix), water penetration-controlled system (swelling, osmotically), chemically controlled system (erodible, pendent), and hydrogel system (diffusion-controlled systems, swelling-controlled system, chemically controlled systems, and environmentally responsive systems) [32]. The different release models represented different release mechanisms. A release model prediction will help researchers find the right track to investigate or modify their samples to a desired controlled release system. The release kinetics models include zero-order release model, which refers to the process of constant drug delivery or matrix tablets with low soluble drugs; first-order release model, which refers to the process of no change in the shape of the solid during the dissolution test or porous matrices with water-soluble drugs; Hixson–Crowell release model, which refers to the process of a change in surface area and diameter or proportionally diminishing of tablets; Higuchi release model, which refers to the process to the simple laws of diffusion or the combination of the zero-order release model and the first-order release model; and Korsmeyer–Peppas release model, which refers to the process of a polymeric system [22,24].

In order to locate the absorption peak of the degradable food colorant, Beckman DU520 UV/Vis spectrophotometer (Beckman Coulter, Brea, CA) was used to perform a 1 nm wavelength sweep. The absorption peaks for the red food degradable colorant ten times diluted aqueous solutions were 504 and 530 nm by red-40 and red-3. In order to evaluate the release percentage, Synergy Neo2 microplate reader (BioTek, Winoski, VT, USA) was used to access the acquired sampled at each time point with absorption at 504 and 530 nm. Within each plate, standard samples (triplicate) were prepared for plate reading. Based on Beer–Lambert’s law, the standard curves were calculated individually for each plate to ensure the consistency of the sampling condition within the plates.

**Figure 6:**
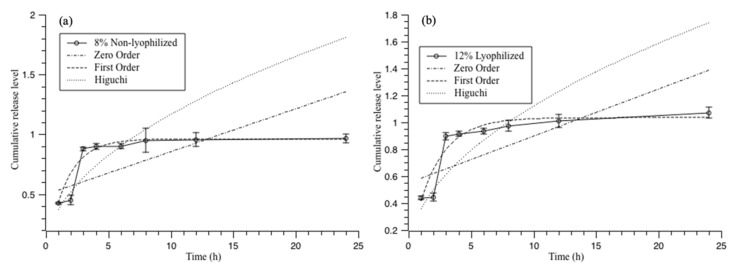
Release model fitting for a standard dissolution test (pH switching at the second hour): (**a**) 8% non-lyophilized CMC hydrogel under 504 nm; and (**b**) 12% lyophilized CMC hydrogel under 530 nm.

From Table 1, compared with the zero-order equation and the Higuchi equation, the first-order equation showed the highest R-square value within non-lyophilized samples and lyophilized samples’ dissolution behavior, indicating that the dissolution process was dominated by a process with no change in the solid or porous matrices with soluble drugs. All the samples showed the lowest R square value in the Higuchi equation and excluded the Higuchi release profile in this study. However, most of the R square value was under 0.9. The highest one in the non-lyophilized samples and overall samples was the non-lyophilized 8%, which showed 0.96 under the first-order equation in 504 nm (red-40) wavelength reading (Figure 6a). The average R square value between the reading of red-40 and red-3 of the non-lyophilized also indicated that 8% of the CMC aqueous hydrogel was capable of a hydrophilic material release profile. The lyophilized samples’ highest R square value was the lyophilized at 12%, which showed that it was at a rate of 0.92 under the first-order equation in 530 nm (red-3) wavelength reading (Figure 6b). The average R square value between the reading of red-40 and red-3 of the lyophilized also indicated 12% CMC aqueous hydrogel was capable for the hydrophilic material release profile. As expected, the results were with a lower CMC concentration (8%) with a non-lyophilization feature that made the saturated capsule more solid-like, which made no-change during the dissolution test. With a higher CMC concentration (12%), the lyophilization post-processing could cause the capsule to be more solid-like as the first-order release model refers to the process of no change in the solid shape during the dissolution test. Above all, the results showed that the 8% CMC aqueous hydrogel without lyophilization could achieve the same function as the lyophilized 12% CMC aqueous hydrogel; both can be used as a capsule for drug delivery use under the first-order release model.

**Figure 7:**
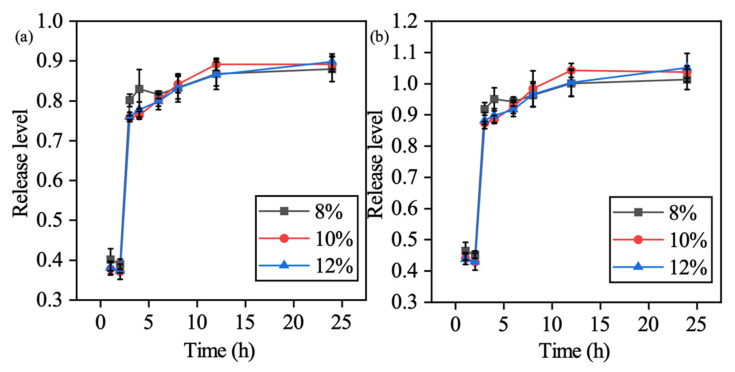
Cumulative release level for the standard dissolution test (pH switching at the second hour) of the lyophilized sample: (**a**) 504 nm and (**b**) 530 nm.

**Figure 8:**
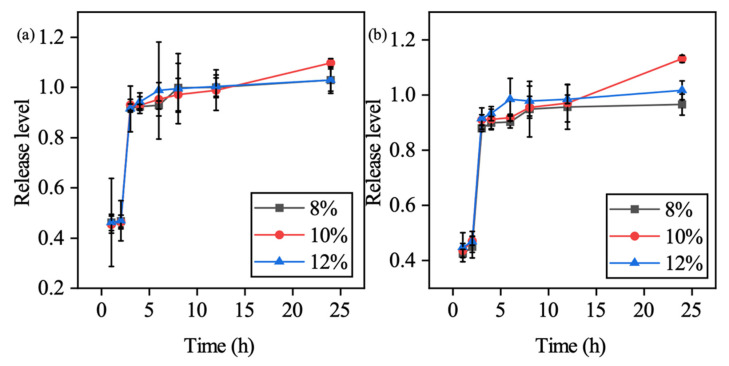
Cumulative release level for the standard dissolution test (pH switching at the second hour) of the non-lyophilized sample: (**a**) 504 nm, and (**b**) 530 nm.

Compared to the non-lyophilized samples, due to the loss of water, and lyophilized samples swelled water in the solution in the beginning, which is why the results showed a more delayed release compared to the non-lyophilized samples. Results showed that within a pH = 1 environment, the colorant release was suppressed due to the integrity preservation under the acidic environment. Upon switching to a pH = 7 environment, the cumulative release can reach a maximum plateau 2 h after environmental changes, which fitted the first-order release model as expected. As shown in Figure 7 the conclusion can be made that with lyophilization post-processing which suppresses the dissolution impact caused by the differentiation of polymer content, the colorants release level which reached 100% at the 24 h time point regardless of the CMC percentage when formulating the hydrogel. However, the non-lyophilized samples showed that a different percentage of CMC hydrogels presented different abilities releasing the colorants in 24 h (Figure 8).

## 5. Conclusions

This study demonstrated the preparation of CMC aqueous hydrogel with lyophilization post-processing. The samples were successfully 3D printed without deformation. The cumulative release profile showed that an 8% non-lyophilized CMC aqueous hydrogel had the same capability as a 12% lyophilized CMC aqueous hydrogel to achieve a no shape change of the solid during the dissolution test with the food colorant. In addition, the lyophilized CMC aqueous hydrogel capsules showed full dissolvability in 24 h. However, non-lyophilized CMC aqueous hydrogel capsules showed different dissolvability under 24 h, compared with lyophilized CMC aqueous hydrogel capsules. The standard dissolution test showed that the CMC aqueous hydrogel had potential application in the pharmaceutical field as a targeted drug delivery system for intestinal delivery. This study could pave the path for the future work of SSE cost-effectively printing drug delivery systems.

## 6. Patents

“Cellulose Derivative Based Biodegradable Support Structures for 3D Printing”, Approved at Iowa State University Office of Intellectual Property and Technology Transfer Office (ISURF 04987), provisional application filed and approved for US Patent.

## Figures and Tables

**Table 1:** R-square value results of release model fitting.

		Zero-Order Model		First-Order Model		Higuchi Model	
CMC %	Wavelength	Non-Lyophilization	Lyophilization	Non-Lyophilization	Lyophilization	Non-Lyophilization	Lyophilization
8	504	0.39034905	0.48813441	0.9592987	0.85242592	0.36952187	0.5348747
10	504	0.58273114	0.49129531	0.86269848	0.81052361	0.45650565	0.34067544
12	504	0.5272996	0.41533232	0.88521804	0.89801676	0.24596937	−0.0187975
8	530	0.21204131	0.50842099	0.75179267	0.84067689	−0.5735051	0.46366171
10	530	0.51079008	0.23943026	0.77361972	0.75222391	0.38693773	–1.3676172
12	530	0.35421321	0.46153722	0.83338572	0.92251983	−1.4281945	0.39575727

## Data Availability

The authors confirm that the data supporting the findings of this study are available within the article.
